# Metabolomics Profiling Reveals the Role of PEDF in Triple-Negative Breast Cancer Cell MDA-MB-231 under Glycaemic Loading

**DOI:** 10.3390/pharmaceutics15020543

**Published:** 2023-02-06

**Authors:** Raziyeh Abooshahab, Kourosh Hooshmand, Giuseppe Luna, Hani Al-Salami, Crispin R. Dass

**Affiliations:** 1 Curtin Medical School, Curtin University, Bentley 6102, Australia; 2 System Medicine, Steno Diabetes Center Copenhagen, 2730 Herlev, Denmark; 3 Biotechnology and Drug Development Research Laboratory, Curtin Health Innovation Research Institute, Bentley 6102, Australia; 4 Curtin Health Innovation Research Institute, Bentley 6102, Australia

**Keywords:** breast cancer, cancer metabolism, metabolomics, glycaemia, PEDF

## Abstract

Pigment epithelium-derived factor (PEDF) is a secreted glycoprotein that belongs to the serine protease inhibitor (serpin) family. An increase in PEDF activity has been shown to be a potent inhibitor of tumour progression and proliferation, suggesting a possible therapeutic target. There is still a great deal to learn about how PEDF controls metabolic pathways in breast cancer and its metastatic form. Given this, the primary purpose of this study was to use a metabolomics approach to gain a better understanding of the mechanisms driving the reprogramming of metabolic events involved in breast cancer pertaining to PEDF under various glycaemic loads. We employed gas chromatography–quadrupole mass spectrometry (GC-Q-MS) to investigate metabolic changes in the triple-negative breast cancer (TNBC) cell line MDA-MB-231 treated with PEDF under glycaemic loading. Multivariate and univariate analyses were carried out as indicative tools via MetaboAnalyst (V.5.0) and R packages to identify the significantly altered metabolites in the MDA-MB-231 cell line after PEDF exposure under glycaemic loading. A total of 61 metabolites were found, of which nine were selected to be distinctively expressed in MDA-MB-231 cells under glycaemic conditions and exhibited differential responses to PEDF (*p* < 0.05, VIP > 1). Abnormalities in amino acid metabolism pathways were observed. In particular, glutamic acid, glutamine, and phenylalanine showed different levels of expression across different treatment groups. The lactate and glucose-6-phosphate production significantly increased in high-glucose vs. normal conditions while it decreased when the cells were exposed to PEDF, confirming the positive influence on the Warburg effect. The TCA cycle intermediates, including malate and citric acid, showed different patterns of expression. This is an important finding in understanding the link of PEDF with metabolic perturbation in TNBC cells in response to glycaemic conditions. Our findings suggest that PEDF significantly influenced the Warburg effect (as evidenced by the significantly lower levels of lactate), one of the well-known metabolic reprogramming pathways in cancer cells that may be responsive to metabolic-targeted therapeutic strategies. Moreover, our results demonstrated that GC-MS-based metabolomics is an effective tool for identifying metabolic changes in breast cancer cells after glycaemic stress or in response to PEDF treatment.

## 1. Introduction

The most common cancer diagnosed in women worldwide is breast cancer (BC). In 2020, there were approximately 2.26 million BC cases and 685,000 BC-related deaths worldwide, with predictions of reaching 3.03 million and 1.04 million by 2040, respectively, according to the Global Cancer Observatory (GCO) [1], making BC the second leading cause of cancer death among females. As a heterogeneous disease, BC is both molecularly and clinically complex [2]. Several classification systems have been proposed for human BC. An example of this is an approach to classifying BC into subtypes based on gene expression patterns determined through complementary DNA microarrays and hierarchical clustering analyses [3]. Luminal-like tumours, including luminal-A (estrogen receptor (ER+)/progesterone receptor (PR+)/oncogene Erb-B2 (HER2−) and luminal-B (ER+/PR−/HER2+) tumours, are the most prevalent subtypes of BC, comprising 40% and 20% of BC cases, respectively, with good prognoses [4,5]. HER2-enriched BCs are responsible for 12% to 20% of all BC cases, representing cancers with overexpression of the Erb-B2 oncogene with a worse prognosis. Basal-like tumours (triple-negative breast cancer (TNBC)) are heterogeneous subtypes that account for 15% of all BCs [4,5]. TNBC lacks the expression of ER and many genes related to ER expression, with a poor prognosis and limited treatment options [6]. TNBC does not respond to the usual treatments for BC, such as hormone therapy or drugs targeting HER2. These tumors tend to be more aggressive and have a higher rate of recurrence than other subtypes of BC [7]. Despite the advances in BC treatment, the management and treatment efficiency is still dismal, particularly when it comes to TNBC conditions. Hence, it is imperative to explore novel and safe therapeutic interventions.

One of the hallmarks of cancer cells is the heightened metabolic activity that boosts cell growth and proliferation [8]. Cancer cells show high demand for energy by heavily relying on glucose, which can be converted to lactate even under oxygen-rich conditions—a phenomenon called the Warburg effect; by doing so, adenosine triphosphate (ATP) is supplied to sustain tumour cell growth and proliferation [9]. Moreover, under hypoxic (low oxygen tension) conditions, ATP is reduced intracellularly in pathophysiological states, leading to rising glucose uptake and aerobic glycolysis metabolism; subsequently, the lactate generation from pyruvate is increased [10]. Over the past decades, growing evidence has shown that hyperglycaemia could affect breast tumour formation and progression, triggering tumour metabolic reprogramming, the Warburg effect, and therapy resistance in breast tumours [11,12]. Hence, developing strategic therapies that specifically affect breast tumours requires comprehensive knowledge concerning the types of metabolic reprogramming in this domain.

Pigment epithelium-derived factor (PEDF) is a 50 kDa secreted glycoprotein that belongs to the serine protease inhibitor (serpin) family encoded by the *SERPINF1* gene [13]. PEDF is a multifunctional protein and possesses differentiating, neurotrophic, antiangiogenic, antiapoptotic, and antimetastatic capabilities [14]. PEDF is generally expressed in human tissues, fluctuating during pathophysiological conditions, including in metabolic syndromes [15], aging-related diseases [16], and cancer [14]. In the wake of the discovery that PEDF modulates the lipolytic pathway through its binding to adipose triglyceride lipase (ATGL) and also improves insulin resistance, it has recently been regarded as a metabolic regulator protein [17]. However, there are still conflicting suggestions as to whether PEDF causes or exacerbates inefficient metabolism. Several studies have been conducted on BC concerning PEDF expression, which showed that PEDF was downregulated in BC, particularly metastatic BC cells [18,19,20,21]. Despite the research on the antitumour and antimetastatic activities of PEDF, little is known about its exact molecular mechanism of tumour growth and progression in response to glycaemic loading, and subsequently the metabolic responses when tumour cells are exposed to PEDF.

In the context of cancer, metabolomics can be used to identify changes in the metabolism of cancer cells that may be exploited for diagnosis, prognosis, or predicting a response to treatment [22]. To date, no metabolomics approaches have been used to investigate how PEDF affects the abundance distribution of metabolites in BC cells, especially under different glucose conditions. In this area, this study aimed to deepen our understanding of the complex mechanisms underlying the reprogramming of metabolic pathways involved in TNCB cells pertaining to PEDF under various glycaemic loads. The TNBC cell line MDA-MB-231 is primarily used in in vitro studies worldwide as it represents a couple of major BC phenotypes, and is a highly invasive and metastatic breast cancer cell line, making it an excellent sample for studying the biology of breast cancer and testing potential therapeutics. Moreover, in MDA-MB-231, the production of ATP is mainly based on glycolysis (Warburg effect), which is a good reason to choose it as a candidate for investigating the landscape of metabolic pathways contributed to tumorigenesis and progression, and subsequently the metabolic responses to the PEDF.

## 2. Materials and Methods

### 2.1. Reagents

The recombinant PEDF was purchased from MD Bioproducts (Bethesda, MD, USA). The cell line (MDA-MB-231) was obtained by the American Tissue Culture Collection, ATCC (Manassas, VA, USA). The media and supplements obtained from Sigma-Aldrich included Dulbecco’s modified Eagle’s medium (DMEM), foetal bovine serum (FBS), and the antibiotics and antimycotics. The isopropanol alcohol (IPA), methanol (MeOH), water (H_2_O)-grade HPLC, undecane (C11), pentadecane (C15), heptadecane (C17), henicosane (C21), pentacosane (C25), C29 nonacosane (C29), tritriacontane (C33), 4,4′-dibromooctafluorobiphenyl, and hexane were purchased from Sigma-Aldrich. The methoxyamine (MOX) and trifluoroacetamide (MSTFA) plus 1% trimethylsilyl chloride (TMCS) reagents were obtained from ThermoFisher Scientific (Waltham, MA, USA).

### 2.2. Cell Line and Culture Conditions

The human BC cell line MDA-MB-231 was initially cultured in normal-dose glucose (5 mM) with DMEM containing 10% heat-inactivated fetal bovine serum (FBS) and 1% antibiotics and antimycotics. All cultures were maintained at 37 °C in a humidified incubator at 5% CO_2_ and passaged 2 times per week following protocols approved by the ATCC. To perform the metabolomics analysis, the MDA-MB-231 cells were seeded in 24-well plates at a density of 3.5 × 10^4^ cells/well in two groups of media containing 5 mM and 25 mM glucose and incubated overnight. The cells were then treated with 100 nM PEDF (a normal physiological concentration of PEDF) [23] and incubated for 24 h. For the intracellular metabolite analysis, the cells were trypsinised and centrifuged at 700× *g* for 5 min. The cell pellets were then stored at 80 °C until sample preparation. Four replicates per media condition were cultivated.

### 2.3. Sample Extraction and Derivatisation

The metabolite extraction process was carried out as follows. A cooled extraction solvent of 2:2:1 (*v*/*v*/*v*) methanol/isopropanol/water was added to each sample tube. The mixtures were vortexed for 60 s, followed by chilling for 20 min at 20 °C, then centrifuged at 21,952× *g* for 15 min at 4 °C. After centrifugation, the supernatants were evaporated to dryness using a 24-position MICROVAP evaporator supplied with nitrogen.

In the first step of the derivatisation process, dried samples were methoximated by adding 30 μL of methoxyamine hydrochloride and mixed for 30 s. Then, the mixtures were placed on a thermo-shaker at 900 rpm for 1 h at 60 °C, followed by the addition of MSTFA containing 1% TMCS and a standard mixture (C11, C15, C17, C21, C25, C29, and C33) of the alkane retention index (50 μL), then placed on a thermo-shaker to react at 900 rpm for 20 min at 45 °C. Afterwards, the samples were mixed with 20 µL of an injection standard that contained 4,4′-dibromooctafluorobiphenyl (concentration = 10 mg/L in hexane). The extracted supernatants were then transferred into a GC-MS autosampler in glass vials and analysed via GC-MS.

### 2.4. GC-Q MS Analysis

Derivatised samples were analysed on an Agilent 5977B MSD/Agilent 8860 GC system equipped with a Restek Rxi-5-ms column (30-m length × 0.25-mm internal diameters (id); 0.25 μm film). Every sample (1 µL) was injected into the inlet at a split ratio of 1:1. Using helium as the carrier gas, the chromatographic method was run at a constant flow rate of 1 mL/min, ramping from 20 °C/min to 320 °C before holding at 320 °C for 5 min. The transfer line, the quadrupole temperature, and the MS source were set at 290, 250, and 150 °C, respectively, in electron ionisation mode at −70 eV. Mass spectrometry data were collected at *m*/*z* 50–600 at a scan rate of 20 spectra/s after a 5.4 min solvent delay.

### 2.5. Data Processing and Statistical Analysis

For data processing, MS-DIAL (version 4.9) was used for peak detection, deconvolution, gap filling, and accurate mass/retention time (*m*/*z*-RT) data. GC-MS spectra were annotated to metabolite names using two orthogonal parameters, such as mass spectral similarity and retention indices. In order to calculate the retention indices, a mixture of alkanes was used. In ChIKey, peak intensity features and the original dataset’s average retention time (RT) were exported from MS-DIAL for a further analysis. A blank subtraction was performed to verify that only features with a maximum sample intensity/average blank intensity ratio greater than 3 were selected. The GC-MS spectra were compared to mass spectral libraries, including Fiehn library, MassBank, Golm DB, GNPS, and the Human Metabolome Database (HMDB). To achieve a normal distribution, the peak intensity was normalised by the sum and scaled using autoscaling in MetaboAnalyst 5.0. Multivariate statistics and visualisation were carried out using a supervised partial least square discriminant analysis (PLS-DA). In the univariate one-way ANOVA, significant differences in mean values were assessed among the different extraction methods. Tukey’s HSD using the R statistics language (ver. 3.5.3) was used to compare significant differences. The R packages “ggpubr” and “tidyverse” were used to visualise the data. To investigate the main biological pathways, we performed an enrichment pathway analysis using MetaboAnalyst (v5.0) with significance thresholds of a *p*-value < 0.05 and FDR < 0.1.

## 3. Results

### 3.1. Untargeted Metabolomics Profiling

We analysed the metabolomic profiles of the human TNBC MDA-MB-231 cell line cultured under two glucose conditions (5 mM for normal glucose and 25 mM for high glucose) exposed to PEDF. As a result of the MS-DIAL data processing, 435 GC-MS peaks were detected, of which 61 metabolites were structurally annotated and were visually reliable across four groups (Table S1), including the normal-glucose and high-glucose groups with and without PEDF.

### 3.2. Effect of PEDF on MDA-MB-231 Metabolomes under Glycaemic Loading

**Figure 1:**
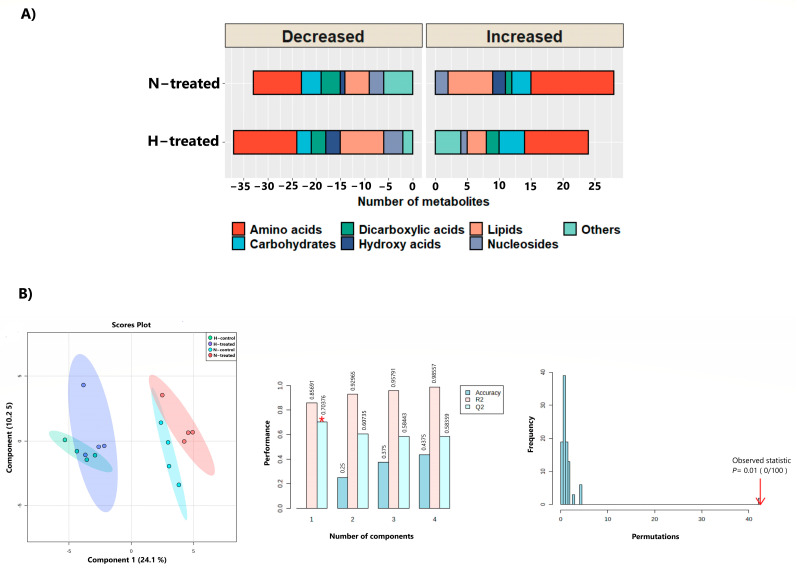
(**A**) Numbers of up- and downregulated metabolites following treatment with PEDF (100 nM, 24 h). (**B**) PLS-DA plot of MDA-MB231 cells under glycaemic loading with and without PEDF created with 95% confidence and cross-validated R2X, R2Y, and Q2 coefficients (middle). The red asterisk implies the best classifier. The histogram showing the permutation test with a permutation number *n* = 100, which showed that the model was significant. ***Key:*** H, high glucose; N, normal glucose.

The large majority of the identified metabolites fell into the categories of amino acids, lipids, and carbohydrates. Dicarboxylic acids, hydroxy acids, nucleotides, and others were less displayed (Figure 1A). The PLS-DA plot revealed a separation between clusters of MDA-MB231 cancer cell samples under glycaemic loading with and without PEDF with good prediction power (Figure 1B, left, middle). The model’s appropriateness was cross-validated by a permutation test (*n* = 100), revealing that the model was significant (Figure 1B, right). The PLS-DA variable importance in projection (VIP) score was determined and features with a VIP score >1 were considered important for group separation (Figure S1).

**Figure 2:**
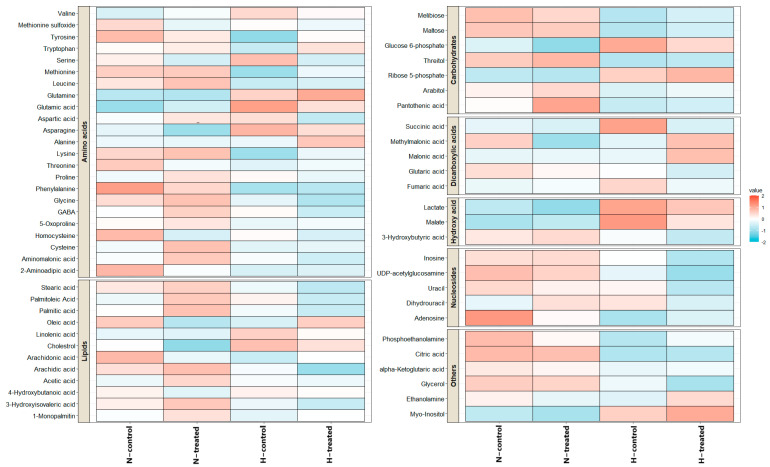
The heatmap shows how the metabolites vary in normal and high-glucose condition with and without PEDF. Identified metabolites were compared based on their average intensities. Red colours indicate increased values, while blue colours indicate decreased values. ***Key:*** H, high glucose; N, normal glucose.

As depicted in Figure 2, the average peak intensities of the metabolomics dataset were pictured as heatmaps to visualise the alterations in metabolites between groups. According to the data, there was a specific pattern of differences in metabolites when cells in the high-glucose and normal-glucose conditions were exposed to PEDF.

**Figure 3:**
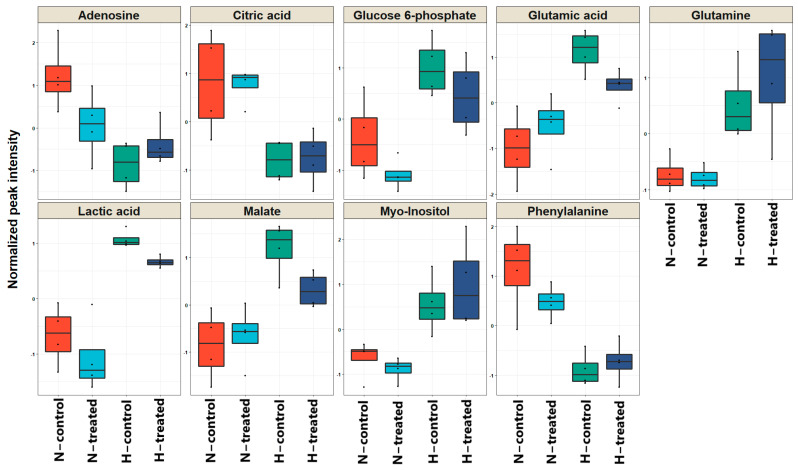
Boxplots of the nine most significant metabolites (*p* < 0.05, VIP > 1) in the analysis of variance results comparing the four groups normal and high-glucose conditions with and without PEDF. The x-axis represents the specific metabolite, and the y-axis is the normalised peak intensity. ***Key*:** H, high glucose; N, normal glucose.

The calculated VIP scores and a one-way ANOVA test were used to find the key metabolites that were significantly altered among the four groups (Table 1). A total of 9 metabolites out of 61 with the criteria of VIP scores >1 and *p*-values of <0.05 were identified as differing significantly between the compared groups. These comprised the TCA cycle and glycolysis intermediates, amino acids, and nucleotides. Figure 3 compares the average intensities for the most significantly altered metabolites among the four groups.

Three amino acids, including glutamic acid, glutamine, and phenylalanine, showed different levels when the cells were cultured in normal and high-glucose conditions. Concomitant with a significant increase in the levels of glutamine in cancer cell lines, the level of glutamic acid was significantly elevated in the high-glucose conditions compared to low-glucose conditions (Figure 3). These differences were more evident for glutamic acid. In addition, after exposing the cells to PEDF, the levels of glutamine moderately decreased and considerably increased in normal and high-glucose conditions, respectively. In contrast, the levels of glutamic acid in normal glucose conditions increased and those in high-glucose conditions decreased substantially when cell lines were exposed to PEDF. Moreover, a decreased level of phenylalanine was observed in high-glucose conditions compared to normal-glucose conditions, and following the PEDF treatment the intensity levels decreased and increased in the normal and high-glucose conditions, respectively. The results showed that the PEDF functioned differently under the two different glucose conditions with respect to the amino acid metabolism.

However, the intensity of the lactic acid substantially increased in high-glucose conditions vs. normal-glucose conditions, but in both high-glucose and normal-glucose conditions the lactic acid intensities decreased after being exposed to PEDF. The level of the major intermediate in glycolysis, glucose 6-phosphate, was also reduced in both conditions with PEDF treatment. These results may indicate the positive effect of PEDF on the Warburg effect, regardless of the glucose level.

TCA cycle intermediates, including malate and citric acid, showed different patterns in terms of their intensities in normal and high-glucose conditions and after exposing the cells to PEDF. Adenosine and myo-inositol were the other significant metabolites that responded differently to PEDF depending on the glucose level. The noteworthy point is that the normal-glucose and high-glucose conditions showed different trends in fatty acid intensities, and PEDF induces different responses, albeit not significantly (Figure 2 and Table S1).

### 3.3. Pathway Analysis

**Figure 4:**
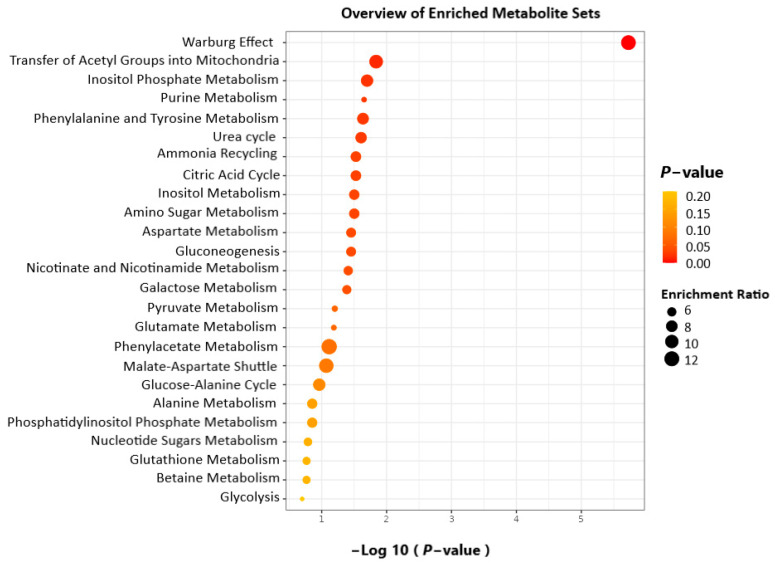
A metabolite set enrichment analysis (MSEA) of affected metabolites pointing out their physiological relevance.

**Figure 5:**
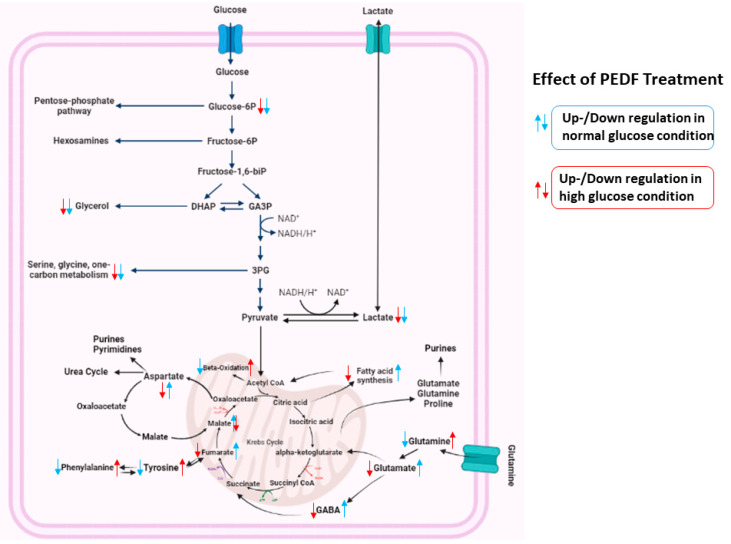
Schematic illustration of the metabolic reprogramming in TNBC cells under glycaemic conditions following PEDF treatment. The Warburg effect, amino acid metabolism, and TCA cycle are all shown as part of the metabolic changes during the PEDF treatment.

An enrichment analysis was performed on the 9 significant metabolites using MetaboAnalyst 5.0. The results indicated that the Warburg effect and the transfer of acetyl groups to mitochondria were the major metabolic factors responsible (Figure 4). The enrichment analysis table containing all the enriched pathways is provided in Table S2. An overview of the altered metabolites exposed to PEDF, and the linked pathways is illustrated in Figure 5. From the results, metabolic reprogramming is characterised mainly by metabolites related to amino acid metabolism, the Warburg effect, and TCA cycle intermediates.

## 4. Discussion

Over the past decades, hyperglycaemia, a metabolic characteristic of diabetes, has been considered one of the most prominent confounding comorbidities and risk factors in the development of BC [11]. Besides being associated with higher mortality and prevalence in BC, hyperglyacemia negatively impacts the functionality of chemotherapy and can lead to chemoresistance [12]. Indeed, reprogramming in glucose metabolism contributes to biological and biochemical pathways by promoting remodelled glycolysis and intensifying acidity uniquely in the tumour microenvironment, consequently enhancing tumour metastasis and recurrence and ultimately leading to the therapeutic resistance of BC [12]. Developing strategic therapies that specifically affect the remodelled pathways in BC requires comprehensive knowledge concerning the types of metabolic reprogramming in this domain.

PEDF is known to be associated with several metabolic disorders linked to insulin resistance, including type 2 diabetes, obesity, metabolic syndrome (PCOS), and hepatic dysfunction [15]. Several studies have demonstrated that PEDF was downregulated in BC, particularly metastatic BC cells [19,20,21]. However, there is no evidence to date suggesting a role of PEDF in the metabolic reprogramming of BC cells under glycaemic conditions. Cancer cell metabolic pathways can be precisely characterised using the metabolomics approach. We used GC/MS-based metabolomics to obtain the altered metabolite profiles in one of the aggressive subtypes of a human BC cell line (MDA-MB 231). Cancer cells utilise glycolysis to provide ATP for their growth and survival (Warburg effect) [24], which is a good reason to choose them as candidates for investigating the metabolic responses to the PEDF treatment under glycaemic loading. To the best of our knowledge, this is the first study that identifies pathways and metabolite level changes in BCs comparing glycaemic conditions treated with PEDF.

Our data showed cell-line-specific responses to PEDF under normal and high-glucose conditions. The high-glucose group showed increased levels of lactate and glucose-6-phosphate compared to the normal glucose group, and their intensity levels were considerably reduced by PEDF, suggesting that the Warburg effect was affected. The Warburg effect refers to a remodelling of glucose metabolism in cancer, where glucose is converted to lactate even when oxygen is present and mitochondria are functioning properly [9]. Other pathways, such as the pentose phosphate pathway (PPP) and one-carbon metabolism, also transform glucose into critical molecules for cancer progression [25]. Our data showed that the high and normal glucose concentrations produced distinct patterns of ribose-5-phosphate (R5P) intensities. The rate of R5P increases in cells when grown under high-glucose conditions compared to normal-glucose conditions. However, these changes were not significant, even though R5P showed a VIP score greater than 1, which contributed towards the discrimination of the PLSD-DA model representing a discriminator metabolite (Table S1). Nevertheless, we did not observe a significant change in this mechanism, but the more we investigate it in the future, the more apparent it should become as to how PEDF might attenuate it.

Cancer cell proliferation is tightly linked with the mitochondrial metabolism [26]. Amino acids may be used as substrates for the TCA cycle in cancer cells in order to maintain ATP production [27]. One of the metabolic features seen in cancer cells is the perturbation of glutamine metabolism. In addition, to generate continuous energy, cancer cells have a high demand for glutamine as a precursor to synthesise other molecules critical for cancer growth and progression [28]. Upon exposure to PEDF, we observed significant alterations to glutamine and glutamic acid levels under glycaemic conditions. Compared to normal conditions, cells in hyperglycaemic conditions contained more glutamine and glutamic acid. Importantly, the levels of glutamic acid in high-glucose conditions without PEDF were significantly increased compared to glutamine. After exposing the cells to PEDF, the glutamine levels increased in high-glucose conditions and decreased in normal glucose conditions. In contrast, glutamic acid was reduced under high-glucose conditions with PEDF and increased under normal-glucose conditions. This shows the different functions of PEDF in normal and high-glucose conditions regarding the metabolism of glutamine and glutamic acid to control the cells’ energy. Glutamine and glutamic acid play a vital role in metabolism and are the primary nitrogen and carbon sources for amino acids, lipids, nucleic acids, and glutathione biosynthesis [29]. The transport of glutamine into cells is followed by its conversion to glutamic acid by an enzyme called glutaminase. Then, glutamic acid is transformed to α-ketoglutarate (α-KG) by glutamic acid dehydrogenase (GDH), which is then supplied to the TCA cycle as fuel [30]. In hyperglycaemic conditions, such as those that occur in diabetes, the levels of these amino acids in the body may be affected. Some studies have suggested that high blood sugar levels may lead to an increase in the breakdown of glutamine in the body to glutamic acid, which could lead to an increase in the levels of glutamic acid [31,32,33]. Additionally, the glutamine and glutamic acid levels may also be influenced by their synthesis and uptake in the body. Hence, this finding, while preliminary, suggests that PEDF has a dual function in high- and low-glucose environments to control the glycaemic conditions by affecting the glutamine and glutamic acid metabolism. The levels of other metabolites, including citric acid and malate, changed in the control and high-glucose conditions. High glucose levels resulted in a decrease in citric acid and an increase in malate. When exposed to PEDF, the cystic acid levels were unaffected but the malate levels showed a slight reduction in high-glucose conditions and an increase in normal-glucose conditions. Thus, a link, albeit weak, may exist between PEDF and mitochondrial metabolism. Phenylalanine is one of the aromatic amino acids whose catabolism contributes to sustaining tumour growth and proliferation. In MDA-MB-231 cells, the phenylalanine levels were high and low in normal and high-glucose conditions, respectively. The intensity of phenylalanine changed by treating BC cells with PEDF, which led to an increase in phenylalanine in high-glucose conditions and a decrease in normal conditions. The phenylalanine metabolism was perturbed in obesity, insulin resistance, and pre-diabetes conditions [34]. Moreover, decreased levels of phenylalanine have been observed in a few tumour types [35,36]. Although the role of phenylalanine in regulating insulin signalling and glucose uptake is unclear, a study by Zhou et al. reported that phenylalanine modifies insulin receptor beta (IRβ) and inactivates insulin signalling and glucose uptake [37].

Another important factor that changed considerably between low- and high-glucose conditions was adenosine, which decreased significantly in high-glucose conditions. After treating cells with PEDF, adenosine decreased and increased in normal and high-glucose conditions, respectively (*p* > 0.05). Adenosine is a nucleoside that is present in all living cells and is involved in many important biological processes, including energy metabolism [38,39]. In cancer cells, adenosine is often found at elevated levels and is thought to play a role in cancer cell metabolism [40]. One way that adenosine may be involved in cancer metabolism is through its role in the generation of ATP (adenosine triphosphate), which is the primary source of energy for cells. Adenosine is converted to ATP through a process called cellular respiration, which occurs in the mitochondria of cells. While cancer cells may have an increased reliance on ATP production through cellular respiration, which may contribute to the abnormal metabolism characteristic of cancer cells [41], the role of adenosine in cancer metabolism is not fully understood. In hyperglycaemic conditions, such as those that occur in diabetes, adenosine may play a role in regulating blood sugar levels. An increased glucose concentration was associated with decreased adenosine levels [42,43]. One way that adenosine may be involved in regulating blood sugar levels is through its effects on insulin secretion. Some studies have suggested that adenosine may stimulate the secretion of insulin, which could help to lower blood sugar levels under hyperglycaemic conditions [44,45]. It can, therefore, be assumed that PEDF has a dual effect on adenosine levels in TNBC cells under glycaemic conditions and control glucose metabolism and the growth of the cells.

Lipid metabolism is a critical facet of tumour progression. It is well established that cancer cells have a higher demand for cholesterol than normal cells and that they often upregulate the synthesis and uptake of cholesterol to meet this demand. This increased demand for cholesterol may be due, in part, to the fact that cancer cells have a higher rate of proliferation and require more cholesterol for membrane biogenesis [46,47]. PEDF exhibited a dual effect on lipid metabolism in MDA-MB-231 cells under normal and high-glucose conditions. In our study, the cholesterol was increased more under high-glucose than normal conditions. Following the PEDF treatment, the cholesterol levels decreased under both conditions. The cholesterol VIP score was higher in the PLS-DA model, which was one of the discriminatory metabolites. It is, therefore, likely that PEDF could modify the lipid metabolism when cells are facing critical states. This warrants further studies in the future.

## 5. Conclusions

Overall, PEDF showed dual effects across the two different glycaemic conditions. The novel findings here point out the potential roles that PEDF plays in BC metabolism and identified novel metabolic markers activated or downregulated by PEDF in TNBC, and showed us the metabolic perturbation of BC cells in two different conditions (normo- and hyperglycaemic), which can possibly assist with developing novel targets for treating patients with triple-negative BC. The GC-MS-based metabolomics approach identified several breast-cancer-specific metabolic traits and demonstrated that the top enriched classes of metabolites were linked to the Warburg effect—the transfer of acetyl groups to mitochondria. This may show how these pathways are disrupted in conditions with various glucose levels to accelerate cancer progression and metastasis, and how PEDF influences them. In view of the diverse metabolism of BC, one limitation of our study is the use of a single cell line to explain the effects of the PEDF treatment on the glycaemic load. To demonstrate the effect of PEDF on cancer metabolism and possibly extend it to other subtypes of cancer, further experiments are needed across multiple BC lines. Furthermore, the effectiveness of PEDF under glycaemic loads requires further research at the protein level to integrate the results. This may be aided to develop novel targets for treating BC patients. Nevertheless, this study has seminally shown the effects of PEDF on metabolites in a TNBC cell line under normal-glucose versus hyperglycaemia conditions.

## Figures and Tables

**Table 1:** Significantly altered metabolites among groups in normal and high-glucose conditions with and without PEDF using a one-way ANOVA and Tukey’s HSD tests.

Metabolites	RT	VIP	One-Way ANOVA		Multiple Comparisons Tukey HSD (^a^ *p*-Value)					
			^a^*p*-Value	FDR	HT vs. HC	NC vs. HC	NT vs. HC	NC vs. HT	NT vs. HT	NC vs. NT
**Lactic acid**	5.58	1.9908	3.63 × 10^−5^	0.002216	0.488	0.0002 ↓↑	4.20 × 10^−5^ ↓↑	0.002 ↓↑	0.0003 ↓↑	0.56
**Phenylalanine**	9.56	1.532	0.00052	0.015849	0.983	0.001 ↑↓	0.025 ↑↓	0.001 ↑↓	0.047 ↑↓	0.304
**Malate**	8.65	1.7187	0.001395	0.023962	0.213	0.001 ↓↑	0.003 ↓↑	0.055	0.121	0.966
**Glutamic acid**	9.46	1.6052	0.001571	0.023962	0.285	0.001 ↓↑	0.006 ↓↑	0.026 ↓↑	0.171	0.68
**Glucose-6-phosphate**	13.01	1.834	0.00251	0.030615	0.601	0.031 ↓↑	0.0024 ↓↑	0.248	0.021	0.481
**Myo-inositol**	13.5	1.5331	0.003189	0.03242	0.794	0.087	0.037 ↓↑	0.017 ↓↑	0.007 ↓↑	0.957
**Glutamine**	10.33	1.4553	0.00443	0.035565	0.735	0.067	0.054	0.011 ↓↑	0.009 ↓↑	0.999
**Citric acid**	10.62	1.5986	0.004664	0.035565	0.999	0.030 ↑↓	0.035 ↑↓	0.032 ↑↓	0.038 ↑↓	0.999
**Adenosine**	14.29	1.1194	0.006105	0.041381	0.785	0.009 ↑↓	0.399	0.046 ↑↓	0.898	0.148

***Key*:** ANOVA, analysis of variance; RT, retention time; VIP, variation important in the projection; FDR, false discovery rate; HT, high-glucose treated; HC, high-glucose control; NT, normal-glucose treated; NC, normal-glucose control. ^a^ *p*-values are from the one-way ANOVA and the Tukey post hoc test; *p* < 0.05 was considered statistically significant. Arrows (↑↓) show up/down regulation of the metabolites that changed significantly.

## Data Availability

All data generated or analysed during this study are included in this article and its Supplementary Materials Files.

## Supplementary Materials

The following supporting information can be downloaded at: https://www.mdpi.com/article/10.3390/pharmaceutics15020543/s1. Figure S1: Variable importance in projection (VIP) scores indicate the most important metabolites that contribute to the separation of metabolic profiles among groups. Table S1: List of identified metabolites extracted from MS-DIAL. Table S2 The mean value of the significant metabolites’ peak intensity. Table S3: The pathway enrichment analysis of altered metabolites.

## References

[1] Ferlay J., Ervik M., Lam F., Colombet M., Mery L., Piñeros M., Znaor A., Soerjomataram I., Bray F. (2020). Global Cancer Observatory: Cancer Tomorrow.

[2] Turashvili G., Brogi E. (2017). Tumor heterogeneity in breast cancer. Front. Med..

[3] Eisen M.B., Spellman P.T., Brown P.O., Botstein D. (1998). Cluster analysis and display of genome-wide expression patterns. Proc. Natl. Acad. Sci. USA.

[4] Sørlie T., Perou C.M., Tibshirani R., Aas T., Geisler S., Johnsen H., Hastie T., Eisen M.B., van de Rijn M., Jeffrey S.S. (2001). Gene expression patterns of breast carcinomas distinguish tumor subclasses with clinical implications. Proc. Natl. Acad. Sci. USA.

[5] Pereira A., Siegrist J., Lizarraga S., Pérez-Medina T. (2022). Clustering Molecular Subtypes in Breast Cancer, Immunohistochemical Parameters and Risk of Axillary Nodal Involvement. J. Pers. Med..

[6] Dent R., Trudeau M., Pritchard K.I., Hanna W.M., Kahn H.K., Sawka C.A., Lickley L.A., Rawlinson E., Sun P., Narod S.A. (2007). Triple-negative breast cancer: Clinical features and patterns of recurrence. Clin. Cancer Res..

[7] Arruebo M., Vilaboa N., Sáez-Gutierrez B., Lambea J., Tres A., Valladares M., González-Fernández Á. (2011). Assessment of the evolution of cancer treatment therapies. Cancers.

[8] Pavlova N.N., Thompson C.B. (2016). The emerging hallmarks of cancer metabolism. Cell Metab..

[9] Warburg O. (1956). On the origin of cancer cells. Science.

[10] Michiels C. (2004). Physiological and pathological responses to hypoxia. Am. J. Pathol..

[11] Vigneri P., Frasca F., Sciacca L., Pandini G., Vigneri R. (2009). Diabetes and cancer. Endocr. -Relat. Cancer.

[12] Qiu J., Zheng Q., Meng X. (2021). Hyperglycemia and chemoresistance in breast cancer: From cellular mechanisms to treatment response. Front. Oncol..

[13] Becerra S.P., Sagasti A., Spinella P., Notario V. (1995). Pigment Epithelium-derived Factor Behaves Like a Noninhibitory Serpin: NEUROTROPHIC ACTIVITY DOES NOT REQUIRE THE SERPIN REACTIVE LOOP (∗). J. Biol.Chem..

[14] Abooshahab R., Al-Salami H., Dass C.R. (2021). The increasing role of pigment epithelium-derived factor in metastasis: From biological importance to a promising target. Biochem. Pharmacol..

[15] Carnagarin R., Dharmarajan A.M., Dass C.R. (2015). PEDF-induced alteration of metabolism leading to insulin resistance. Mol. Cell. Endocrinol..

[16] Abooshahab R., Dass C.R. (2021). The biological relevance of pigment epithelium-derived factor on the path from aging to age-related disease. Mech. Ageing Dev..

[17] Huang K.-T., Lin C.-C., Tsai M.-C., Chen K.-D., Chiu K.-W. (2018). Pigment epithelium-derived factor in lipid metabolic disorders. Biomed. J..

[18] Palmieri D., Fitzgerald D., Shreeve S.M., Hua E., Bronder J.L., Weil R.J., Davis S., Stark A.M., Merino M.J., Kurek R. (2009). Analyses of Resected Human Brain Metastases of Breast Cancer Reveal the Association between Up-Regulation of Hexokinase 2 and Poor PrognosisUp-Regulation of HK2 Is Associated with Brain Metastasis. Mol. Cancer Res..

[19] Zhou D., Cheng S.-Q., Ji H.-F., Wang J.-S., Xu H.-T., Zhang G.-Q., Pang D. (2010). Evaluation of protein pigment epithelium-derived factor (PEDF) and microvessel density (MVD) as prognostic indicators in breast cancer. J. Cancer Res. Clin. Oncol..

[20] Fitzgerald D.P., Subramanian P., Deshpande M., Graves C., Gordon I., Qian Y., Snitkovsky Y., Liewehr D.J., Steinberg S.M., Paltán-Ortiz J.D. (2012). Opposing Effects of Pigment Epithelium–Derived Factor on Breast Cancer Cell versus Neuronal Survival: Implication for Brain Metastasis and Metastasis-Induced Brain Damage. Cancer Res..

[21] Hong H., Zhou T., Fang S., Jia M., Xu Z., Dai Z., Li C., Li S., Li L., Zhang T. (2014). Pigment epithelium-derived factor (PEDF) inhibits breast cancer metastasis by down-regulating fibronectin. Breast Cancer Res. Treat..

[22] Schmidt D.R., Patel R., Kirsch D.G., Lewis C.A., Vander Heiden M.G., Locasale J.W. (2021). Metabolomics in cancer research and emerging applications in clinical oncology. CA A Cancer J. Clin..

[23] Petersen S.V., Valnickova Z., Enghild J.J. (2003). Pigment-epithelium-derived factor (PEDF) occurs at a physiologically relevant concentration in human blood: Purification and characterization. Biochem. J..

[24] Gatenby R.A., Gillies R.J. (2004). Why do cancers have high aerobic glycolysis?. Nat. Rev. Cancer.

[25] Cho E.S., Cha Y.H., Kim H.S., Kim N.H., Yook J.I. (2018). The pentose phosphate pathway as a potential target for cancer therapy. Biomol. Ther..

[26] Porporato P.E., Filigheddu N., Pedro J.M.B.-S., Kroemer G., Galluzzi L. (2018). Mitochondrial metabolism and cancer. Cell Res..

[27] Wei Z., Liu X., Cheng C., Yu W., Yi P. (2021). Metabolism of amino acids in cancer. Front. Cell Dev. Biol..

[28] DeBerardinis R.J., Cheng T. (2010). Q’s next: The diverse functions of glutamine in metabolism, cell biology and cancer. Oncogene.

[29] Cha Y.J., Kim E.-S., Koo J.S. (2018). Amino acid transporters and glutamine metabolism in breast cancer. Int. J. Mol. Sci..

[30] Altman B.J., Stine Z.E., Dang C.V. (2016). From Krebs to clinic: Glutamine metabolism to cancer therapy. Nat. Rev. Cancer.

[31] Felig P., Wahren J., Karl I., Cerasi E., Luft R., Kipnis D.M. (1973). Glutamine and glutamate metabolism in normal and diabetic subjects. Diabetes.

[32] Li P.-A., Shuaib A., Miyashita H., He Q.-P., Siesjö B.K. (2000). Hyperglycemia enhances extracellular glutamate accumulation in rats subjected to forebrain ischemia. Stroke.

[33] Stumvoll M., Perriello G., Meyer C., Gerich J. (1999). Role of glutamine in human carbohydrate metabolism in kidney and other tissues. Kidney Int..

[34] Adams S.H. (2011). Emerging perspectives on essential amino acid metabolism in obesity and the insulin-resistant state. Adv. Nutr..

[35] Miyagi Y., Higashiyama M., Gochi A., Akaike M., Ishikawa T., Miura T., Saruki N., Bando E., Kimura H., Imamura F. (2011). Plasma free amino acid profiling of five types of cancer patients and its application for early detection. PLoS ONE.

[36] Yuan B., Schafferer S., Tang Q., Scheffler M., Nees J., Heil J., Schott S., Golatta M., Wallwiener M., Sohn C. (2019). A plasma metabolite panel as biomarkers for early primary breast cancer detection. Int. J. Cancer.

[37] Zhou Q., Sun W.-W., Chen J.-C., Zhang H.-L., Liu J., Lin Y., Lin P.-C., Wu B.-X., An Y.-P., Huang L. (2022). Phenylalanine impairs insulin signaling and inhibits glucose uptake through modification of IRβ. Nat. Commun..

[38] Haskó G., Antonioli L., Cronstein B.N. (2018). Adenosine metabolism, immunity and joint health. Biochem. Pharmacol..

[39] Fredholm B.B., Ijzerman A.P., Jacobson K.A., Klotz K.N., Linden J. (2001). International Union of Pharmacology. XXV. Nomenclature and classification of adenosine receptors. Pharmacol. Rev..

[40] Leone R.D., Emens L.A. (2018). Targeting adenosine for cancer immunotherapy. J. Immunother. Cancer.

[41] Fernandez-Gallardo M., González-Ramírez R., Sandoval A., Felix R., Monjaraz E. (2016). Adenosine stimulate proliferation and migration in triple negative breast cancer cells. PLoS ONE.

[42] Sakowicz M., Szutowicz A., Pawelczyk T. (2005). Differential effect of insulin and elevated glucose level on adenosine transport in rat B lymphocytes. Int. Immunol..

[43] Phillis J.W., Simpson R., Walter G. (1990). The effect of hyperglycemia on extracellular levels of adenosine in the hypoxic rat cerebral cortex. Brain Res..

[44] Dong Q., Ginsberg H., Erlanger B. (2001). Overexpression of the A1 adenosine receptor in adipose tissue protects mice from obesity-related insulin resistance. Diabetes Obes. Metab..

[45] Vannucci S.J., Nishimura H., Satoh S., Cushman S.W., Holman G., A Simpson I. (1992). Cell surface accessibility of GLUT4 glucose transporters in insulin-stimulated rat adipose cells. Modulation by isoprenaline and adenosine. Biochem. J..

[46] Mayengbam S.S., Singh A., Pillai A.D., Bhat M.K. (2021). Influence of cholesterol on cancer progression and therapy. Transl. Oncol..

[47] Ding X., Zhang W., Li S., Yang H. (2019). The role of cholesterol metabolism in cancer. Am. J. Cancer Res..

