# Supplementary material for: Metabolomics Profiling Reveals the Role of PEDF in Triple-Negative Breast Cancer Cell MDA-MB-231 under Glycaemic Loading
Source: Pharmaceutics. 2023 Feb 6;15(2):543. doi: 10.3390/pharmaceutics15020543 (PMC9962752; doi:10.3390/pharmaceutics15020543)
Supplement: Supplementary file 1 [file pharmaceutics-15-00543-s001.zip › pharmaceutics-2180790-supplementary.pdf]

Metabolomics profiling reveals the role of PEDF on triple-negative breast cancer cells under glycaemic loading

Raziyeh Abooshahab [1], Kourosh Hooshmand [2], Giuseppe Luna [1], Hani Al-Salami [1,3] and Crispin R. Dass [1,4,*]

[1] Curtin Medical School, Curtin University, Bentley 6102, Australia
[2] System Medicine, Steno Diabetes Center Copenhagen, 2730 Herlev, Denmark;
[3] Biotechnology and Drug Development Research Laboratory, Curtin Health Innovation Research Institute, Bentley 6102, Australia
[4] Curtin Health Innovation Research Institute, Bentley 6102, Australia
* Correspondence: crispin.dass@curtin.edu.au; Tel.: +61-8-9266-1489

Supplemental Table S1 List of identified metabolites extracted from MS-DIAL

Supplemental Table S2 The mean value of the significant metabolites' peak intensity

Supplemental Table S3 Pathway Enrichment analysis of altered metabolites

Supplemental Figure S1 Variable importance in projection (VIP) scores indicate the most important metabolites that contribute to the separation of metabolic profiles among groups

Table S1. List of identified metabolites extracted from MS-DIAL.

Metabolite name	Average Rt(min)	Average RI	Quant mass	Class
**1-Monopalmitin**	14.005	2585.56	371.3	Lipids and lipid-like molecules
**2-Aminoadipic acid**	10.042	1729.86	73	Organic acids and derivatives
**3-Hydroxyisovaleric acid**	6.742	1223.26	131.0133	Lipids and lipid-like molecules
**4-Hydroxybutanoic acid**	6.992	1258.62	116.975	Organometallic compounds
**Acetic acid**	12.399	2209.06	69.05	Lipids and lipid-like molecules
**Adenosine**	14.296	2655.68	230.0444	Nucleosides, nucleotides, and analogues
**alpha-Ketoglutaric acid**	9.143	1574.42	73.1	Organic acids and derivatives
**Aminomalonic acid**	8.574	1482.19	147.02	Organic acids and derivatives
**Arachidic acid**	13.395	2440.7	369.32	Organometallic compounds
**Arachidonic acid**	13.162	2386.52	80	Lipids and lipid-like molecules
**Cholesterol**	17.227	3265.5	129.0063	Lipids and lipid-like molecules
**Citric acid**	10.623	1843.8	73.025	Organic acids and derivatives
**Cysteine**	9.109	1568.8	69.06	Organic acids and derivatives
**D-Arabitol**	10.114	1743.83	217.0778	Organic oxygen compounds
**Dihydrouracil**	6.138	1137.92	56.02	Organoheterocyclic compounds
**3-Hydroxybutyric acid**	6.27	1156.51	191.0636	Organic acids and derivatives
**DL-Homocysteine**	9.752	1676.77	234.0583	Organic acids and derivatives
**L-5-Oxoproline**	8.923	1537.46	73.06	Organic acids and derivatives
**D-Ribose 5-phosphate**	12.116	2143.26	315.0143	Organic oxygen compounds
**D-Threitol**	8.82	1520.24	73.1	Organic oxygen compounds
**Ethanolamine**	7.192	1286.86	174.0813	Organometallic compounds
**Fumaric acid**	7.583	1342.11	81.95	Organic acids and derivatives
**GABA**	8.954	1542.65	174.1	Organic acids and derivatives
**Glucose 6-phosphate**	13.103	2372.75	387.0616	Organic oxygen compounds
**Glutaric acid**	8.085	1413.03	84.06667	Organic acids and derivatives
**Glycerol**	7.263	1296.93	191.0125	Organometallic compounds
**Glycine**	7.488	1328.65	73.05833	Organic acids and derivatives
**Inosine**	14.046	2595.37	73.06	Nucleosides, nucleotides, and analogues
**Malate**	8.66	1494.41	73.01538	Organic acids and derivatives
**Phenylalanine**	9.568	1645.82	218.1	Organic acids and derivatives
**Proline**	7.423	1319.49	142.1	Organic acids and derivatives
**Threonine**	8.023	1404.33	73.0625	Organic acids and derivatives
**Lactic acid**	5.578	1044.26	73.05	Organic acids and derivatives
**Lysine**	11.145	1946.16	174.1	Organic acids and derivatives
**Alanine**	5.919	1106.96	116.0875	Organic acids and derivatives
**Asparagine**	9.802	1685.19	231.025	Organic acids and derivatives
**Aspartic acid**	8.246	1435.88	101.96	Organic acids and derivatives
**Glutamic acid**	9.464	1628.37	247.1	Organic acids and derivatives
**Glutamine**	10.359	1792.02	155.975	Organic acids and derivatives
**Linolenic acid**	12.508	2234.34	80	Lipids and lipid-like molecules
**L-Leucine**	7.093	1272.9	231.0769	Organic acids and derivatives
**Methionine**	8.889	1531.85	176.075	Organic acids and derivatives

**Serine**	7.835	1377.68	173	Organic acids and derivatives
**Tryptophan**	12.62	2260.43	202.0538	Organoheterocyclic compounds
**Tyrosine**	11.247	1966.03	218.1	Organic acids and derivatives
**Malonic acid**	6.476	1185.6	73.1	Organic acids and derivatives
**Maltose**	14.665	2744.56	73.04285	Organic oxygen compounds
**Melibiose**	15.052	2837.76	204.0429	Organic oxygen compounds
**Methionine sulfoxide**	10.228	1766.3	128.0364	Organic acids and derivatives
**Methylmalonic acid**	6.612	1204.87	147.04	Organic acids and derivatives
**Myo-Inositol**	11.766	2067.82	147.0333	Organic oxygen compounds
**valine**	6.51	1190.52	86.05	Organic acids and derivatives
**Oleic acid**	12.478	2227.4	75	Lipids and lipid-like molecules
**O-Phosphoethanolamine**	10.45	1809.81	299.0077	Organic acids and derivatives
**Palmitic acid**	11.674	2049.86	73.1	Lipids and lipid-like molecules
**Palmitoleic Acid**	11.569	2029.26	101.96	Organometallic compounds
**Pantothenic acid**	11.518	2019.12	103.0077	Organic acids and derivatives
**Stearic acid**	12.575	2249.89	73	Lipids and lipid-like molecules
**Succinic acid**	7.472	1326.43	247.0667	Organic acids and derivatives
**Uracil**	7.695	1357.99	184.0333	Organoheterocyclic compounds
**Uridine 5'-diphospho-N-acetylglucosamine**	10.449	1809.54	231	Nucleosides, nucleotides, and analogues

RT retention time, RI retention indices

Table S2. The mean value of the significant metabolites' peak intensity.

Metabolites	Groups	Mean
**Adenosine**	N-control	1.207529
	N-treated	0.052681
	H-control	-0.86652
	H-treated	-0.39369
**Citric acid**	N-control	0.808662
	N-treated	0.749081
	H-control	-0.80556
	H-treated	-0.75219
**Glucose 6-phosphate**	N-control	-0.38244
	N-treated	-1.08695
	H-control	1.014286
	H-treated	0.455104
**Glutamic acid**	N-control	-0.99802
	N-treated	-0.49917
	H-control	1.128907
	H-treated	0.368277
**Glutamine**	N-control	-0.73131
	N-treated	-0.7921
	H-control	0.51794
	H-treated	1.005465
**Lactic acid**	N-control	-0.66212
	N-treated	-1.07169
	H-control	1.070445
	H-treated	0.663359
**Malate**	N-control	-0.86021
	N-treated	-0.64782
	H-control	1.190296

	H-treated	0.317733
**Myo-Inositol**	N-control	-0.65143
	N-treated	-0.89268
	H-control	0.548133
	H-treated	0.995974
**Phenylalanine**	N-control	1.139066
	N-treated	0.476861
	H-control	-0.88983
	H-treated	-0.7261

Key: H, high glucose, N, normal glucose

Table S3. Pathway Enrichment analysis of altered metabolites.

Metabolic pathway	[a] Total Cmpd	[b] hits	[c] Raw p	[d] FDR
**Warburg Effect**	58	6	1.88E-06	0.000185
**Transfer of Acetyl Groups into Mitochondria**	22	2	0.0145	0.287
**Inositol Phosphate Metabolism**	26	2	0.02	0.287
**Purine Metabolism**	74	3	0.0222	0.287
**Phenylalanine and Tyrosine Metabolism**	28	2	0.0231	0.287
**Urea Cycle**	29	2	0.0247	0.287
**Ammonia Recycling**	32	2	0.0297	0.287
**Citric Acid Cycle**	32	2	0.0297	0.287
**Inositol Metabolism**	33	2	0.0315	0.287
**Amino Sugar Metabolism**	33	2	0.0315	0.287
**Aspartate Metabolism**	35	2	0.0352	0.287
**Gluconeogenesis**	35	2	0.0352	0.287
**Nicotinate and Nicotinamide Metabolism**	37	2	0.039	0.287
**Galactose Metabolism**	38	2	0.041	0.287
**Pyruvate Metabolism**	48	2	0.0628	0.399
**Glutamate Metabolism**	49	2	0.0651	0.399
**Phenylacetate Metabolism**	9	1	0.0767	0.442
**Malate-Aspartate Shuttle**	10	1	0.0849	0.462
**Glucose-Alanine Cycle**	13	1	0.109	0.562
**Alanine Metabolism**	17	1	0.14	0.655
**Phosphatidylinositol Phosphate Metabolism**	17	1	0.14	0.655
**Nucleotide Sugars Metabolism**	20	1	0.163	0.697
**Glutathione Metabolism**	21	1	0.171	0.697
**Betaine Metabolism**	21	1	0.171	0.697
**Glycolysis**	25	1	0.2	0.767
**Cysteine Metabolism**	26	1	0.207	0.767
**Selenoamino Acid Metabolism**	28	1	0.222	0.767
**Pentose Phosphate Pathway**	29	1	0.229	0.767
**Folate Metabolism**	29	1	0.229	0.767
**Lysine Degradation**	30	1	0.236	0.767
**Starch and Sucrose Metabolism**	31	1	0.243	0.767
**Beta-Alanine Metabolism**	34	1	0.263	0.805
**Propanoate Metabolism**	42	1	0.315	0.9
**Methionine Metabolism**	43	1	0.321	0.9
**Histidine Metabolism**	43	1	0.321	0.9
**Arginine and Proline Metabolism**	53	1	0.381	1
**Glycine and Serine Metabolism**	59	1	0.415	1
**Pyrimidine Metabolism**	59	1	0.415	1
**Valine, Leucine and Isoleucine Degradation**	60	1	0.421	1
**Tryptophan Metabolism**	60	1	0.421	1
**Arachidonic Acid Metabolism**	69	1	0.468	1
**Tyrosine Metabolism**	72	1	0.483	1

[a] Total Cmpd: total number of compounds in the pathway, [b] Hit: actually matched number from the data
[c] Raw p: p-value calculated from the enrichment analysis, [d] FDR: p-value adjusted using False Discovery Rate

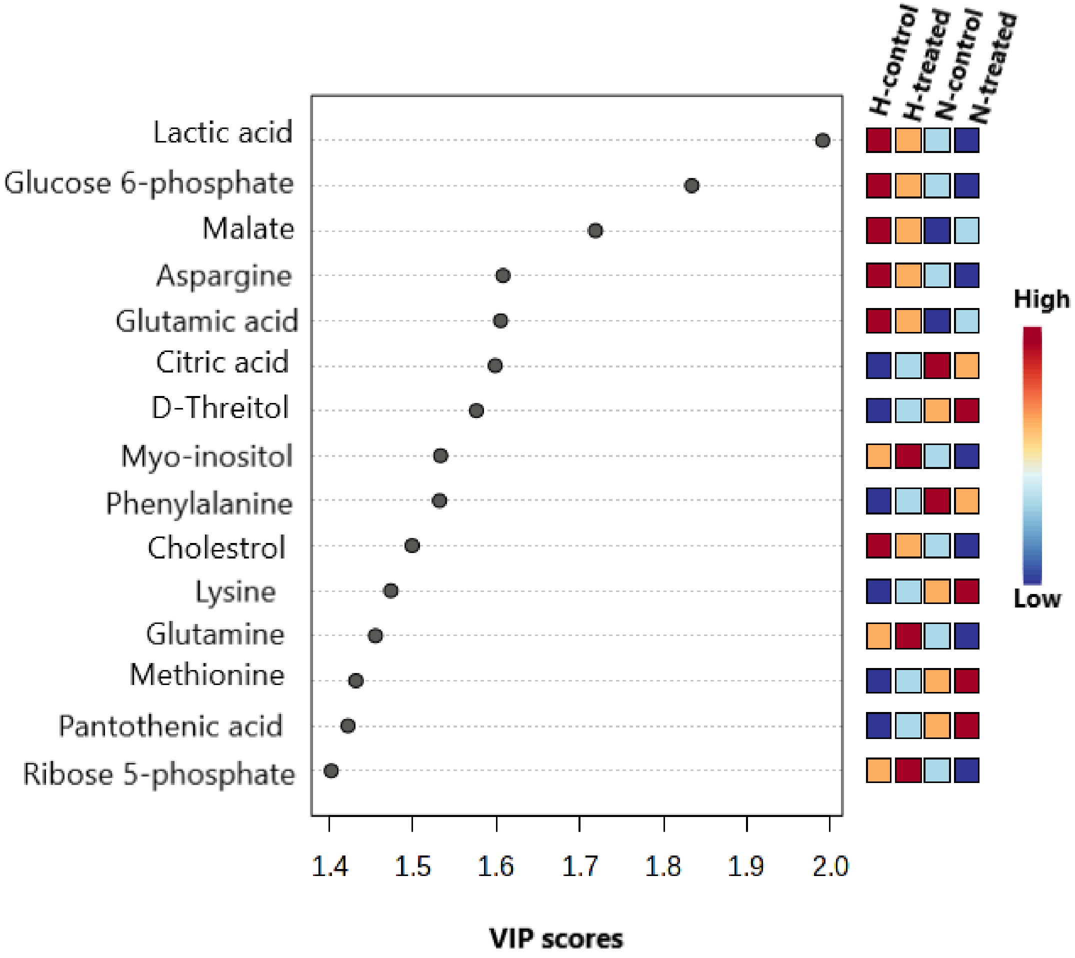

Figure S1. Variable importance in projection (VIP) scores indicate the most important metabolites that contribute to the separation of metabolic profiles among groups. Metabolite abundances are indicated on a colour scale from blue to red, which represents low and high, respectively.
